# Olive shrub buried on Therasia supports a mid-16th century BCE date for the Thera eruption

**DOI:** 10.1038/s41598-023-33696-w

**Published:** 2023-04-28

**Authors:** Charlotte Pearson, Kostas Sbonias, Iris Tzachili, Timothy J. Heaton

**Affiliations:** 1 Laboratory of Tree-Ring Research, University of Arizona, 1215 E. Lowell Street, Tucson, 85721 USA; 2 Geosciences, University of Arizona, 1040 E. 4th Street, Arizona, 85721 USA; 3 Anthropology, University of Arizona, 1009 E. South Campus, Arizona, 85721 USA; 4 Department of History, Ionian University, I. Theotoki 72, 49100 Corfu, Greece; 5 Department of History and Archaeology, University of Crete, A. Papandreou str., 74100 Rethymnon, Greece; 6 Department of Statistics, School of Mathematics, University of Leeds, Leeds, LS2 9JT UK

**Keywords:** Carbon cycle, Natural hazards, Volcanology, Environmental social sciences, Environmental impact

## Abstract

The precise date of the 2nd millennium BCE (“Minoan”) eruption of Thera (Santorini) has long been a focus of controversy due to a discrepancy between archaeological and radiocarbon-based dating of materials from stratigraphic layers above and below tsunami, ash and pumice deposits resulting from the eruption. A critical, though controversial, piece of evidence has been four segments of a radiocarbon-dated olive tree branch, buried on Thera during the eruption. Here we report new radiocarbon evidence from an olive shrub found carbonized by the same eruption deposits on neighboring Therasia (Santorini). The Therasia olive shrub dates slightly younger than the previous olive branch. Calibrated results and growth increment counts indicate increased probabilities for a mid-16th century BCE date for the eruption, overlapping with multiple volcanic sulfate markers from ice core records.

## Introduction

**Figure 1:**
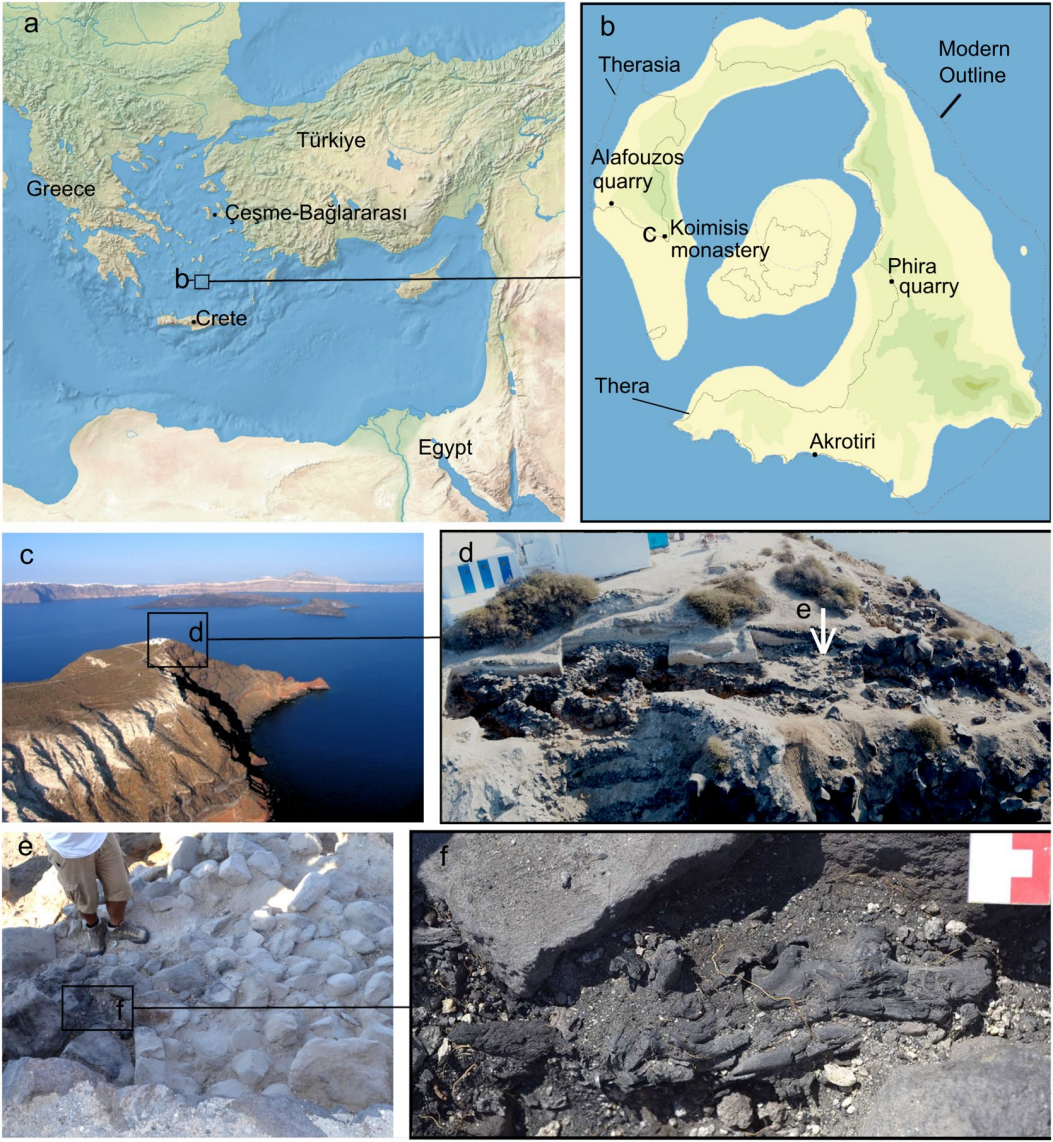
The location of Thera (**a**, **b**), Koimisis (**c**) and the olive shrub (**d**, **e**). Localized burning associated with the olive remains (**e**) and the olive shrub remains in situ (**f**). Image credit: Sbonias.

The eruption of Thera in the Santorini archipelago, Greece, (Fig. 1a,b), sometime in the 2nd millennium BCE, sealed the spectacular Minoan settlement of Akrotiri and other sites on Thera and Therasia under meters of volcanic debris^1,2^. The eruption is thought to have been the largest of the Holocene in terms of volume, and highly explosive, with a Volcanic Explosivity Index (VEI) of 7^3^. It deposited a marker horizon of volcanic material across the wider region, thereby providing a critical synchronization point for the chronologies of the Aegean, Anatolia, the Levant, and Egypt. According to the archaeological chronology of the southern Aegean (based on pottery types) the eruption occurred at the transition between the Late Minoan IA and Late Minoan IB period and was initially speculated to have led to the fall of the Minoan civilization on Crete^4^. The size, impact, and, critically, the exact date of the Thera eruption, have been widely discussed in the literature^5–9^, with persistent differences between the calibrated age distributions based on radiocarbon (^14^C) determinations of materials buried by eruption deposits^9–13^, placing the eruption around 1600 BCE, and archaeologically-based arguments for an eruption date after the start of the New Kingdom in Egypt. While some studies suggest that the New Kingdom could have begun c.1560 BCE^8^ or a decade or so earlier^14^, conventional dating places it ‘after 1540 BCE’, with some arguing for 1524 BCE as an exact date for the eruption^7,15^.

Recent improvements to the IntCal radiocarbon calibration curve^16^, which now includes over 800 high-precision, multi-laboratory ^14^C measurements on calendar dated tree-rings between 1700 and 1500 cal BCE, have gone some way to addressing this gap between radiocarbon and archaeological arguments by providing a state-of-the-art calibration resource which precisely describes the radiocarbon plateau and small inversion c.1620-1540 BCE. The occurrence of the eruption during the plateau period has, however, long posed a serious limitation for radiocarbon dating based on associated short-lived samples. Firstly, during a radiocarbon age plateau it is not possible to obtain a precise calendar age for an event from a single ^14^C determination, since any calendar age on the plateau would be consistent with the observed ^14^C. For events which occur during plateaus, precise dating can be obtained only by modelling multiple ^14^C determinations simultaneously, for example via a wiggle-match^17^ or an appropriate phase model^18^. This introduces some subjectivity as to the appropriate form of the model for the ^14^C determinations. Secondly, the plateau effect means that the presence of even small (c.10 ^14^C year) interlaboratory^19^ or regional^20^ offsets in the radiocarbon determinations for individual samples, plus the size of associated errors, have the potential to change significantly the plausible range of calibrated dates, or substantially shift uncertainties in favour of one calibrated date over another. While the potential effects of this, plus mixed age deposits in archaeological contexts, can be well accounted for within programs such as OxCal^18,21^, CALIB^22^ and CalPal^23^, subjective decisions on whether to model averaged values^24^, which data to include, how to model them, and which archaeological observations to incorporate all have an impact on the end date range, resulting in a number of possible dating placements proposed for essentially the same materials (e.g.^9,13,25,26^).

In 2006, a sample retrieved from an olive tree (hereafter called the Santorini olive) buried under the $>60$-m-thick Minoan pumice layer exposed in the caldera wall^27^ at Phira Quarry (Fig. 1b) appeared to offer a way to mitigate the problematic ^14^C plateau effect and arrive at a more precise and accurate date for the eruption. Trees contain records of radiocarbon collected across multiple years, with the oldest wood at the center and the youngest at the outer edge. This means that the calendar age could be better constrained if the record of ^14^C in the olive branch captured the slope in the calibration curve preceding the plateau. However, this sample has proved highly controversial because olive trees do not necessarily produce annual growth rings in the way that pine or oak trees might^28^. While some studies have shown that annual growth rings in olives are possible^26^, others have shown that the nature of olive growth may mean that a stem or branch with bark may have actually ceased to accumulate growth bands several decades before the death of the tree^29^. As a consequence, it has been recognised^10^ that we are neither able to treat the four dated blocks sampled from the Santorini olive branch as corresponding to a precise known number of annual growth bands, nor consider the reported internal-age gaps between these four blocks as exact. This hinders our ability to wiggle-match the sequence against the calibration curve and brings a subjective element into the modelling. Furthermore, uncertainty exists over whether the part of the branch sampled was still growing when it was buried by the Thera eruption, although the presence of stratified leaves indicates that the tree itself was alive^30^. A simple ordered sequence^12^ (based on the indisputable fact that the inner-most sampled material from the olive is older than the outer-most material) suggests, at 95.4% (2-$\sigma$) probability using IntCal20, that the sample died either between 1621 and 1598 BCE (23.2%) or between 1593 and 1540 BCE (72.3%). This is in general agreement with results from more complex modelling of a wide range of short-lived pre- and post-Theran eruption materials^9^. Without new radiocarbon dating evidence this is the current limit of what is possible for Thera in terms of radiocarbon dating.

In this study, we report radiocarbon dates from a second olive (henceforth called the Therasia olive) found during excavations near the present day Monastery of Panaghia Koimisis, on the southern-most tip of the island of Therasia^2^ at the western side of the present-day Santorini caldera (Fig. 1b–f). At the time of the Minoan eruption, the settlement at Koimisis was in ruins which were buried under the primary ash deposits. In 2016, a trench was opened in an upper terrace of the hill (trench AB) (Fig. 1d,e), where the eruption layers were still partially preserved to a maximum depth of around 90 cm^2^. The charred shrub remains were found immediately below the layer of pumice, in between some terraced stones which showed localized burning (Fig. 1e,f). Samples taken from the shrub were studied using high-resolution microphotograpy at the Laboratory of Tree-Ring Research, University of Arizona, and sampled for radiocarbon dating at the University of Arizona Accelerator Mass Spectrometery laboratory. Dating results were compared with the original Santorini olive and other data from secure Theran contexts in the surrounding region, and with time-series of measurements on securely dated single tree-rings from the same growth region, produced at the same laboratory during the same period of analysis, in order to rule out the possibility of inter-laboratory differences. Finally, the radiocarbon results were evaluated in the context of work to synchronize the global record of volcanic sulfate from polar ice-cores spanning the approximate possible time range for the Thera eruption^31^. This approach has produced a series of calendar or near-calendar dated volcanic sulfate markers between c.1620–1500 BCE. While confirming that Thera was a lower sulfate event (therefore with lower associated climatic forcing potential) the sulfate that it did produce (between 0.34–36 Tg of S^32^) would certainly have entered the stratosphere. The tropopause is c.14 km above sea level at the latitude of Santorini, and both the Plinian^33^ and co-ignimbrite^34^ phases of the eruption are calculated to have produced columns in excess of 30 km, so some sulfate transportation to the ice sheets seems certain. This being the case, one of the dated ice-core sulfate markers in this period likely represents the Thera eruption. The Therasia olive provides an important new opportunity to gain further insights into which of these sulfate signals may be best supported by radiocarbon dating evidence for Thera, and consequently perhaps a new possibility to obtain a precise eruption date.

## Results

### Dendrochronological investigation

**Figure 2:**
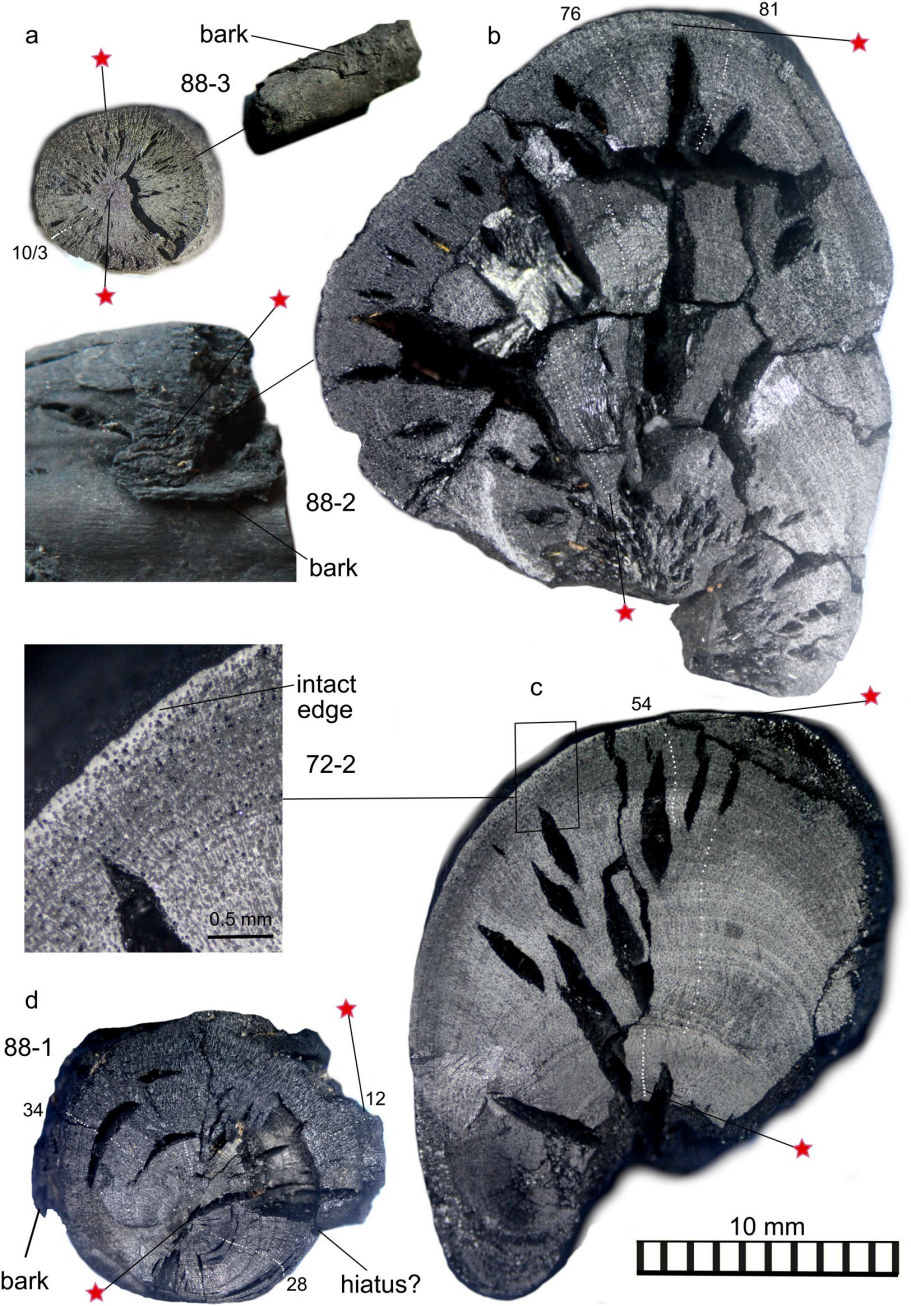
Enlarged photo-micrographs of sub-samples of the Therasia olive shrub showing approximate growth increment counts and inherent difficulties in interpreting growth patterns. Red stars show radiocarbon sampling locations. 88-3 and the bark of 88-2 produced the youngest radiocarbon dates from the shrub as a whole at the time of death. Image credit: Pearson/Siekacz.

The pieces of shrub were 2.5 cm or less at widest diameter in transverse cross-section, with visible bands ranging from c.500 – 80 $\mu$m. These showed some inconsistency around the stem leading to slightly different counts along separate radii of each sample. High resolution photo-micrographs were used to enlarge sample cross-sections for improved counting. Figure 2a–d illustrates the problematic nature of the material, which was further complicated by a number of cracks and fractures. There were, however, sections of incremental growth where band counts were reproducible (e.g. Fig. 2c). We note that these increments do not necessarily represent years of growth^29^, although this could be the case^26^. It was possible to determine ‘waney-edge’ (the outer-most, terminal xylem growth at a given location in the shrub) and / or bark on all sub-samples selected for radiocarbon analysis, although the presence of bark in olive trees does not necessarily indicate that the underlying xylem at the time of tree-death was active^26^. Given the difficulties in accurate sampling (due to the sub-500 $\mu$m banding and brittleness of the material) we took only radiocarbon samples from the inner and outer part of each sub-sample, plus bark from the most securely confirmed outer edge. Notes describing each sample / sub-sample, associated counts and radiocarbon sampling are provided in the methods section.

**Table 1:** Radiocarbon ages (^14^C years BP) for four stem samples taken from the Therasia olive shrub.

Lab Number	Sample ID	^13^C	FM	FM 1$\sigma$	^14^C age	^14^C 1$\sigma$
AA111457	88-3 Inner	− 23.5	0.6619	0.0019	3314	23
AA110275	88-3 Outer	− 22.8	0.6633	0.0019	3297	23
AA110272	88-2 Inner	− 24.4	0.6581	0.0017	3361	21
AA110273	88-2 Outer	− 23.4	0.6598	0.0019	3341	23
AA110274	88-2 Bark	− 24.2	0.6630	0.0018	3301	23
AA111456	88-1 Inner	− 24.6	0.6551	0.0018	3398	21
AA110271	88-1 Outer	− 23.1	0.6615	0.0018	3320	22
AA111458	72-2 Inner	− 24.6	0.6583	0.0019	3358	23
AA111459	72-2 Outer	− 22.8	0.6596	0.0020	3342	24

### Radiocarbon dating

**Figure 3:**
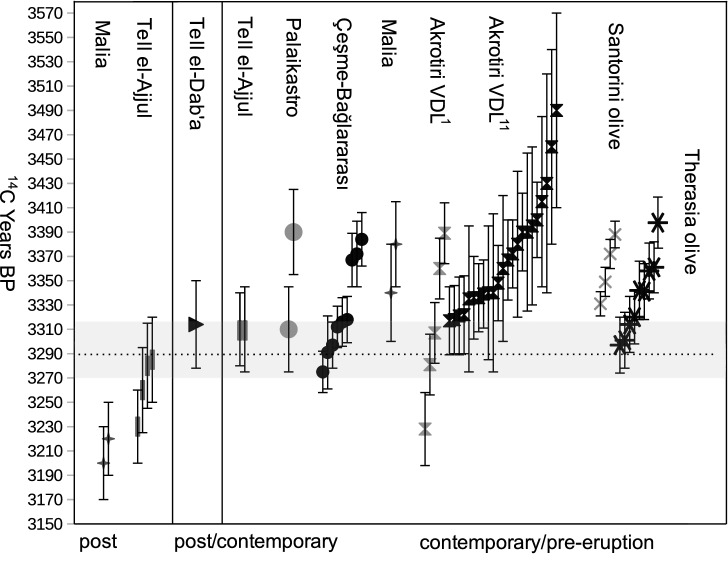
The youngest ${}^{14}$C measurement from the Therasia olive (dotted horizontal line) with stated errors (grey horizontal band) relative to other uncalibrated dates from secure pre- and post-Thera eruption contexts. From left to right: Post-tsunami stratigraphy, Malia, Crete^36^; after pumice arrives at Tell el-Ajjul^37^; after pumice arrives at Tell el-Dab’a^38^; before pumice arrives at Tell el-Ajjul^37^; in tsunami deposits at Palaikastro, Crete^39^ and at Çeşme-Bağlararası, Türkiye^40^; pre-tsunami stratigraphy, Malia, Crete^36^; volcanic destruction layer (VDL) at Akrotiri^1,11^; Phira quarry, Thera^27^, Therasia olive, this study.

The uncalibrated radiocarbon measurements derived from the Therasia olive (Table 1) are broadly consistent with determinations for the Santorini olive^27^. However the outer-most material from the Therasia olive indicates slightly younger ^14^C ages (Fig. 3). A tiny shoot (sample 88-3, Fig. 2a) with an intact, thin, juvenile bark (which would have been rapidly lost after death) was interpreted as likely being alive at the time of carbonization. This hypothesis is supported by the radiocarbon results, with the youngest ^14^C determinations overall produced from the inner and outer-most parts of this sample. These two ^14^C ages also align with a measurement from the bark of sample 88-2. This bark result appears to be c.20 ^14^C years younger than the last formed part of the intact cambium directly underlying it (88-2-outer). While bark may typically represent accumulation of ^14^C over a number of years, the fact that the overall determination for the bark sample is younger than that of the outer-most part of the stem, and is the same as the result for the shoot, indicates that the years represented are cumulatively more recent than the last formed part of the stem. Indeed as olive bark has been shown to possess efficient photosynthetic mechanisms^35^ it would likely have been taking up ^14^C via photosynthesis right up to the time the shrub died (i.e., during the eruption), even if the stem below it had ceased growing.

**Figure 4:**
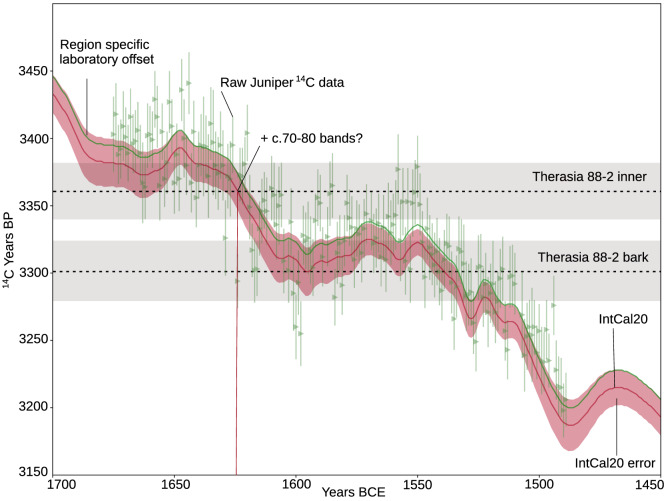
Radiocarbon measurements from single years of a Mediterranean juniper tree (green) run at the same laboratory / same time as the Therasia samples, relative to IntCal20^16^. The offset towards older ages reflects the difference between data from a single region / single laboratory and the IntCal20^16^ mean. To illustrate uncertainties associated with calibration, the inner-most and bark results from stem 88-2 are shown (horizontal dotted line, grey error) intersecting with two radiocarbon plateaus in the time period. If considered to represent annual growth, band counting of sample 88-2, combined with the most likely point of intersection for the inner-most determination with intCal20^16^, indicates a death date towards the middle of the 16th century BCE, but clear uncertainties remain.

Figure 3 shows that the uncalibrated dates for the Therasia shrub’s outer-most bark and shoot samples are not only consistent with one another (3301 / 3297 ± 23 ^14^C years BP), they are also consistent with determinations from short lived materials from the volcanic destruction layer at Akrotiri^11^ and from Theran tsunami deposits at Çeşme-Bağlararası, Türkiye^40^ (e.g., 3297 ± 19 ^14^C years BP) and Palaikastro on Crete^39^, as well as with samples found in contemporary mainland contexts marked by the sudden arrival of Theran pumice^37,38^. These results reinforce that there is no major volcanic ^14^C offset in play on Thera in the years leading up to the eruption. If increased out-gassing of old CO_2_ had been absorbed by the growing shrub one would expect to see a reversal in the ^14^C age gradient of the olive samples, or a rather older set of outer-most ^14^C measurements relative to the ^14^C dates for materials from Çeşme-Bağlararası. The data in this study, as well as for the original Santorini olive^27^, show an old to young gradient in terms of radiocarbon measurement from inner to outer-most sample, with radiocarbon ages approximately supporting growth increment counts. The slightly younger ^14^C ages for the outer-most Therasia samples compared with that of the outer-most part of the Santorini olive^27^ could be explained in a number of ways. This may be real, reflecting a true age difference in the sampled materials and indicating that the outer-most part of the sampled Santorini olive had stopped growing a little before the eruption. It could equally be a product of the pooling of a longer temporal span in the outer-most piece of the Santorini olive, which included at least 13 growth increments representing an unknown ^14^C accumulation period. Alternatively, it may be the result of slight inter-laboratory differences^19^ at the time of analysis of the different samples. Potential laboratory-offsets should also be considered in the interpretation of the agreement between all other data shown (Fig. 3), as should the radiocarbon plateau effect. While it is tempting to comment more generally on the excellent and compelling agreement of this radiocarbon evidence, we note that the radiocarbon plateau means that essentially the same ${}^{14}$C years BP measurement would be produced for any true calendar year between c.1620 and 1540 BCE (see Fig. 4).

### Offset testing prior to calibration

A large sequence of single year ^14^C from Mediterranean juniper trees growing approximately 400 km to the east of Thera during the time of the eruption^41^ was analyzed over a c. 6 month period in random (non-sequential) order at the University of Arizona radiocarbon laboratory. The Therasia samples were interspersed with these samples during the same analytical period (see supplementary information, M1). Plotted relative to IntCal20, these single year juniper ^14^C data can be seen to lie consistently older than the IntCal20 curve (see Fig. 4). A formal Bayesian analysis suggests that the juniper measurements are offset by c. +13.7 ± 2 ^14^C years (See supplementary information, M2). The most likely explanation for this offset is that it represents the difference between the consensus IntCal20 data, derived from trees in different growth locations run at different laboratories, and a single-lab, single region data set.

The IntCal20 construction method^42^ adapts to the possibility that there are some *stochastic* sources of variability in tree-ring ^14^C measurements beyond those quantified as a laboratory’s measurement uncertainty (e.g., variation between laboratories, by growth location, or species). The predictive uncertainties reported on the IntCal20 curve are designed to incorporate the possibility of such unquantified variation also being present in the samples one wishes to calibrate against the curve, so that, when calibrating single ^14^C samples against IntCal20, the possibility of such unquantified variation is automatically accounted for. Indeed, the observed juniper difference is just within the upper predictive interval of IntCal20. However, when jointly calibrating multiple ^14^C samples which are all affected by a *systematic* offset, for example in an ordered sequence or a wiggle match, it is necessary to model that shared offset explicitly during calibration^13^. The consistency of the juniper offset over time (and the potential for even small offsets to have substantial effects during ^14^C plateaus) means that such a *systematic* offset should be considered when jointly calibrating the multiple ^14^C determinations from the Therasia shrub, assuming it may share the same offset as the contemporary juniper data.

**Figure 5:**
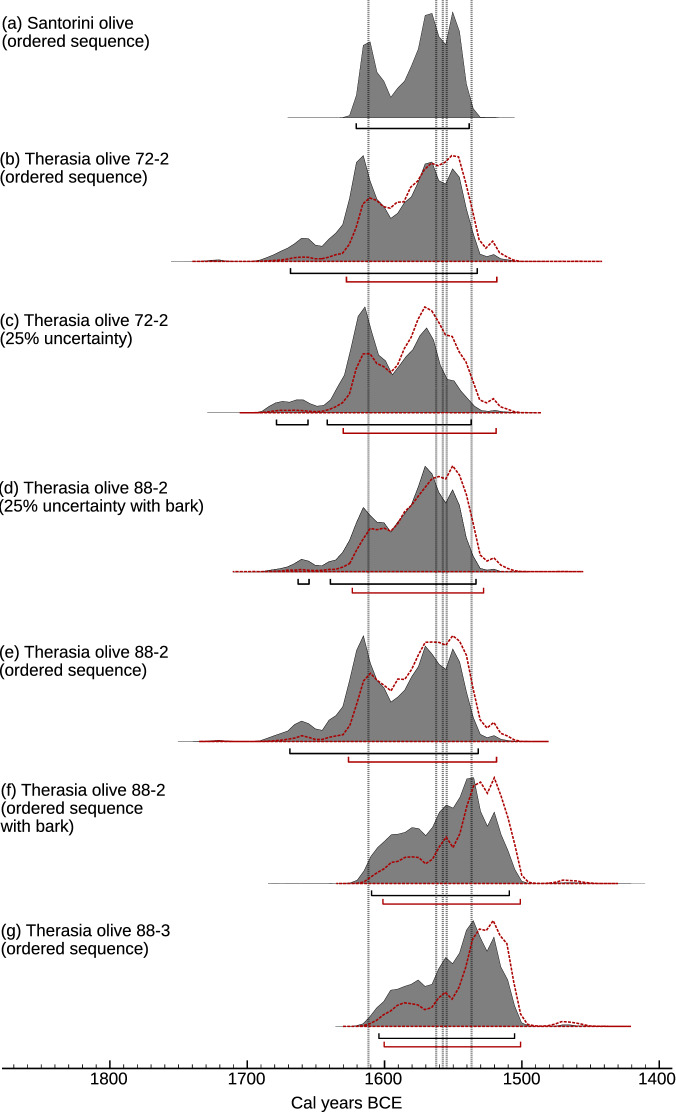
Calibrated dating possibilities for the separate stems of the Therasia olive compared with the Santorini olive (**a**), modelled as a simple ordered sequence (see^12^) relative to IntCal20^16^ using Oxcal^18^ v4.4.4. 72-2 (**b**, **c**) and 88-2 (**d**, **e**) had the clearest growth boundaries so are shown as both an ordered sequence and a V-sequence at 25% uncertainty. 88-2 had an intact outer-most edge with bark which was also dated and is included in (**d**) and (**f**). 88-3, the small shoot is shown as (**g**), noting that the band count between samples in this case was very few. The changes in probability distribution using the juniper-based offset correction are shown with a red dotted outline. Grey vertical lines indicate current key volcanic sulfate layers in the Greenland ice^31^.

For our analyses, we performed two distinct sets of calibrations: the first assumed no offset between the Therasia olive and the IntCal20 curve; the second applied an offset in OxCal of Delta_R(”offset”, 13.7, 2). Which approach provides the more reliable, modelled, calendar ages (those obtained with, or without, the Delta_R offset) will depend upon whether or not the offset from IntCal20 identified in the contemporaneous Mediterranean juniper is truly shared by the olive shrub. Due to the common laboratory factors and latitude we might expect it to be, however other potential issues such as species could not be fully explored. Importantly, however our main conclusions are not substantially affected by whether we include, or exclude, the Delta_R offset during calibration (Fig. 5). This provides confidence that our findings are robust to this modelling choice.

### Calibration

Each sampled stem of the Therasia shrub (72-2, 88-1, 88-2, and 88-3) was calibrated separately. Outer-most samples were not averaged as not all stems automatically had the same time of death; a single shrub can have both living and dead branches which sprouted at different times. Instead, separate results for each part of the shrub were compared to derive the most likely outer date placement representing the shrub at time of death. Given clear uncertainties in terms of counting and defining the units of time represented by the shrub’s growth bands, we advocate caution in the use of these boundaries in modelling, but include tests on a range of scenarios in SI (Table S1, Figs. S1,2,3 and 4 and M3). Sample-72-2 had the clearest defined boundaries, and produced the most reproducible counts. 88-2 produced higher numbers of counts but these were less reproducible. 88-1 appeared to have a hiatus in growth (see Fig. 2) supported by incongruous band counts relative to the ^14^C determinations. This stem also produced the oldest pith date for the shrub, which may indicate that it sprouted before the other samples, or, alternatively may be an instrumental outlier. 88-3 was mostly pith with barely discernible banding. Given the inherent uncertainties associated with even the clearest defined boundaries in the samples, we suggest that a simple ordered sequence model^12^, based on the indisputable fact that the inner (first grown) material from each sample was older than the outer-most (last grown) material from each sample is the most sound basis for each calibration. Modelled in this way, the four increments of the Santorini olive show strong agreement with the various results from the Therasia shrub (Fig. 5), with wide-ranging dating possibilities in both the 17th and 16th centuries BCE.

In terms of their 95.4% (2-$\sigma$) probability intervals, results for samples 88-2 and 88-3 (thought to be representative of the youngest parts of the shrub at time of death) both indicate an outer-most calibrated age range for the shrub of 1610–1510 BCE when no Therasia-specific Delta_R offset from IntCal20 is considered; or, if the juniper-based laboratory/regional offset of +13.7 ± 2 ^14^C years is applied, c.1602–1502 BCE. This 95.4% calendar age range differs only very slightly from those produced for the outer-most segments of the other samples and the Santorini olive (Fig. 5) and from a range of more subjective modelling scenarios tested for the samples (SI, Table S1, Figs. S1,2,3 and 4 and M3). Due to the plateau effect, we would only expect minor differences to this broad 95.4% range: any determination potentially consistent with the plateau will likely have a calibrated 95.4% range that extends over all of it. However, the specific calendar ages with the greatest posterior probability within that broad 95.4% range do shift substantially (Fig. 5, SI, Figs. S1–4 ).

In particular, results from samples 88-2 and 88-3 indicate a much greater posterior age density during the later 16th century BCE (Fig. 5d–g). This result is significant because it suggests an increased likelihood that Thera erupted in the mid to later 16th century BCE, consistent with certain archaeological arguments, including the controversial^9^ 1524 BCE date^7,15^. Caution is necessary however in interpreting the result for 88-3 as it is based on two radiocarbon measurements that are identical within errors, which fall fully on the radiocarbon plateau and are separated by only a few possible growth bands. The result for stem 88-2 on the other hand has the advantage of an inner-most radiocarbon measurement which anchors the sample on an earlier plateau. If this is correct, and the growth increment count (though uncertain) is factored in (Figs. 4, 5d), the calibrated result places the greater posterior age density closer to the mid 16th century BCE.

### Comparison with known, ice core derived dates for volcanic eruptions in the same temporal period

Figure 5 also shows calendar dated ice-core sulfate layers at 1611 BCE, 1562-1555 BCE and c.1538 BCE which have been highlighted as possible markers for the Thera eruption^31^. Two additional volcanic events identified at 1586 BCE (Greenland) and 1550 BCE (Antarctica) are not shown as they are currently considered less likely for Thera based on tephra / hemispheric deposition evidence^31^, but should be further investigated. The sulfate at c.1538 BCE is not as securely dated as the other markers due to brittle ice across this time period. This sulfate could shift to eventually align with previously noted tree-ring climatic marker events in the 1540s^12^. The brittle ice has also meant that it has not yet been possible to asses whether another ice core sulfate exists around c.1524 BCE, when a severe climatic response observed in tree-rings^43^ has been suggested as a potential candidate date for Thera^7,15^. Modelled results (Fig. 5f,g) indicate that this date is worthy of further exploration, but if the observed growth bands in the Therasia olive are indeed representative of something approximating annual growth, this date would not be so well supported (Fig. 5d). Our ^14^C results also do not wholly rule out an association with 1611 BCE sulfate, however the low density of the posterior calendar age estimate on the youngest samples from the Therasia olive, suggests a lower probability that this event is Thera and increases the likelihood that Thera is therefore one of the eruption signals between 1562-1555 or c.1538 BCE.

## Discussion

The calibrated ranges (Fig. 5d–g) that best represent the youngest portion of the Therasia olive, carbonized *in situ* by the Thera eruption, indicate radiocarbon dating possibilities for the eruption encompassing the late 17th and entire 16th century BCE. This reflects once again the problematic nature of attempting to date an event which occurs on a radiocarbon plateau (Fig. 4). However, the increased posterior probability density in the mid to late 16th century (Fig. 5) offers some significant new possibilities when reviewed in the context of recent ice-core data. 1611 BCE, a date considerably older than could be archaeologically supported (assuming that Thera erupts after the start of the New Kingdom in Egypt^7,8,15^), is marginalised by our dating results. Younger sulfate markers on the other hand, at 1562–1555 BCE and c.1538 BCE, coincide with increased posterior calendar age density for the outer-most samples from Therasia and the Santorini olive. Fine tuning models via our offset adjustment of +13.7 ± 2 ^14^C years relative to IntCal20 (as suggested by 181 contemporaneous juniper measurements from the same Mediterranean region measured in the same laboratory and over the same analytic period) further increased the probabilities around these younger events. Further work to define and better understand the growth boundaries in the Therasia samples, combined with additional radiocarbon sampling, may yield additional insights, especially in constraining possibilities with regard the more marginal 1611 BCE and 1524 BCE event dates. This study underlines the limitations of the more subjective aspects of a modelled approach, combined with the effect of the calibration curve plateau and the limitations of existing radiocarbon data available from Theran contexts. Currently these make it impossible to resolve the debate over an exact date for the Thera eruption with radiocarbon evidence alone. Nonetheless, this study improves on previous modelled iterations via the Therasia data and may bring the long running impasse between radiocarbon and archaeological evidence for the dating of Thera closer to a final resolution.

## Methods

### Archaeological context of the shrub

The Koimisis olive shrub was excavated a few meters south of the present day Monastery of Panaghia Koimisis, at an Early to Middle Bronze Age clifftop settlement 196m above sea level at the edge of the caldera^2^. The site sits on lava flows, dated c. 45,000-25,000 years ago, and the ashes of the Cape Riva eruption of Thera c. 22,000 years ago. Erosion of the Minoan eruption deposits on the south-east side of the area revealed terraced slopes predating the eruption, while the north-west part of the site was totally covered by the eruption debris. The remains of the olive shrub were recovered from trench AB, immediately below the Minoan pumice, between some charred stones. The location of the shrub on the pre-eruption surface suggests that it started life sometime after the settlement was abandoned. Death and charring were inferred to be simultaneous as a consequence of burial by the hot ash from Thera. The burnt stones were situated at the top of the pre-eruption surface and did not continue in depth, therefore a rapid fire burnt only what existed on the surface in the vicinity of the charring shrub. The layer of burnt soil (plus a few late Middle Cycladic sherds) was very thin (5-7 cms), below which soil, bone and pottery were all unburnt. Burnt and unburnt sherds from the same broken vessel provided supporting evidence of the superficial nature of the burning.

Based on ceramic evidence, the site at Koimisis parallels the life-span of the Middle Cycladic - Late Cycladic I town of Akrotiri. This town suffered two seismic destructions, one in the late Middle Cycladic period (Middle Cycladic phase C at Akrotiri) with imported Cretan material that dates in the MM IIIA^44^ and one early in the Late Cycladic I period (the so-called ‘Seismic Destruction Level’). These two seismic destructions were chronologically very close^45^. The abandonment of the settlement at Koimisis seems to coincide with this first destruction, as there are no traces of Late Cycladic I pottery which is found at Akrotiri in association with the second seismic event. Sample DEM-1458^1^ from inside a destruction-debris layer in a Late Middle Cycladic building at Akrotiri, directly overlain by a Late Cycladic I building, calibrates to IntCal20 (OxCal 4.4.4) with a multi-modal distribution at 95.4% probability: 1744-1609 cal BCE (92.3%), 1576-1562 cal BCE (2.2%), 1554-1547 cal BCE (0.9%). The oldest measured part of the Therasia shrub (88-1 inner) calibrates to 1741-1617 BCE at at 95.4% probability, consistent with the 92.3% range, supporting a date for the first earthquake during this period. The approximate growth band counts and radiocarbon results are all also broadly consistent with a period of not more than c.100 years between the first destruction and the eruption.

**Table 2:** Description of sub-samples taken from the Therasia olive shrub, growth band counts and associated radiocarbon sampling.

Sample ID	Sample description	Radiocarbon sampling
88-3	8 mm round wood shoot with clear outer edge and pith. c.3-10 growth bands, intact juvenile bark	88-3I = pith; 88-3O = outer edge
88-2	c.2.5 cm twisted stem with 2–3mm bark fixed to intact outer edge. Pith present. c. 76-81 inconsistent growth bands < 400 μm, most visible at outer edge, counts uncertain due to high fracturing and erratic growth	88-2I = c.5 inner-most bands; 88-2O = c.5 outer-most bands; 88-2B = mature outer bark
88-1	c.1 cm, round wood shoot with clear outer edge and pith. c. 40 indistinct, tightly packed fine bands < 400 μm, with an indistinguishable area of growth which may represent a growth hiatus	88-1I = 4 inner-most bands including pith; 88-1O = c. 3 outer-most bands
72-2	c.1.5 cm stem with intact outer edge, pith not present. c.54 inconsistent growth bands < 500 μm, areas of distinct banding where more replicable counts were possible	72-2I = c.4 inner-most bands; 72-2O = 5 outer-most bands

### Dendrochronological analysis and radiocarbon sampling

Sampling in the field focused on the removal of small diameter stems from the main trunk which was too twisted and brittle to sample. All methods used were in accordance with the required archaeological permitting protocols. The stems were selected based upon the presence of bark and any visible banding that might be related to growth. At the Laboratory of Tree-Ring Research, four sub-samples with the best prospects for accurate sampling of an inner and outer segment (with bark or bark edge confirmed) were selected for further analysis. Cross-sections of each sample were prepared using a controlled break, scalpel and fine grade sand-paper to reveal the wood’s internal structure and the complex nature of the growth boundaries. Surfaces were examined and photographed under x20 and x40 magnification, and attempts were made to measure the growth boundaries for each sample using a Velmex measuring platform with Tellervo software^46^. However, due to the small size and poor definition of the growth boundaries and their variation around the stems, this process did not produce usable data. Instead, high resolution composite photomicrographs were produced using a Canon EOS digital SLR camera mounted on a Leica compound microscope, then enlarged for final counting and marked up with dots to allow for reproducibility of sampling and further testing of the assigned boundaries (Fig. 2). Radiocarbon samples from the inner-most / outer-most edge and bark were taken using a scalpel under x10 magnification and put in foil packets for transfer to the University of Arizona AMS Laboratory. Sampling details are provided in Table 2.

### Radiocarbon sample preparation and analysis

The carbonized olive samples were converted to holocellulose at the University of Arizona Accelerator Mass Spectrometry (AMS) Laboratory. This involved standard 1N HCl / NaOH / HCl extractions at 70°C followed by a holocellulose extraction at 70°C using a bleaching solution made from sodium chlorite, HCl and water. Treated samples appeared bright white in color. Samples were combusted to CO_2_ and converted to graphite using standard procedures^47^, then measured using a National Electrostatics Corporation AMS system, operated at a terminal voltage of 2.5 MV. The ^14^C/^13^C ratio of each sample was compared to National Institute of Standards and Technology standards SRM4990B and 4990C and the resulting fractionation corrected to a delta ^13^C value measured offline on a stable isotope mass spectrometer. Results were calibrated using OxCal v.4.4.4^48^ and the IntCal20 calibration curve^16^. Code provided in SI.

## Supplementary Information

Supplementary Information.

## Data Availability

Raw data for this study are published in Table 1. The juniper ^14^C series used to calculate the offset is available in published supplementary data files^41^. Intcal20 data are available through^16^.
