# Supplementary material for: Olive shrub buried on Therasia supports a mid-16th century BCE date for the Thera eruption
Source: Sci Rep. 2023 Apr 28;13:6994. doi: 10.1038/s41598-023-33696-w (PMC10147620; doi:10.1038/s41598-023-33696-w)
Supplement: Supplementary file 1 — Supplementary Information. [file 41598_2023_33696_MOESM1_ESM.pdf]

Supplementary Information:

Olive shrub buried on Therasia supports a mid-late 16th century BCE date for the Thera eruption

Charlotte Pearson

Kostas Sbonias

Iris Tzachili

Timothy J. Heaton

Contents

Table S1: Example calibrated ranges of possible modeled outcomes for the Therasia olive

Fig S1: Range of possible modeled outcomes for sample 72 from the Therasia olive (probability density distributions)

Fig S2: Range of possible modeled outcomes for sample 88-1 from the Therasia olive (probability density distributions)

Fig S3: Range of possible modeled outcomes for sample 88-2 from the Therasia olive (probability density distributions)

Fig S3: Range of possible modeled outcomes for sample 88-3 from the Therasia olive (probability density distributions)

Supplementary methods

M1: Details of off-set justification

M2: Details of off-set calculation

M3: Details of modeling choices

Table S1: Extended modeling possibilities for the various pieces of the Therasia Olive. Samples 88-3 (Bark) and 88-2 (inner and outer) are considered to represent the youngest parts of the shrub at time of death, other stems may represent slightly different start and end dates in terms of growth or may be essentially the same in terms of radiocarbon error possibilities. This table shows that choices in the way the data are modeled make very little difference to the over-all span of the possible date range for the death of the shrub, as would be predicted given the radiocarbon plateau. The differences in probability distributions can be seen in figures S1,2,3 and 4.

“OS” = simple ordered sequence, “NB” = no bark, “UN” = 25% uncertainty applied, “WB”= with bark included, “OFF” = offset applied.

Test	Model	Range	Bi-modal distribution
722_OS_NB	Ordered Sequence	1670 – 1533	
722_OS_NB_OFF	Ordered Sequence	1628 – 1518	
722_UN_NB	25% Uncertainty	1681 – 1539	(1681 – 1658, 1644 – 1539)
722_UN_NB_OFF	25% Uncertainty	1631 – 1519	
722_WM_NB	Wigglematch	1684 – 1547	(1684 – 1666, 1642 – 1547)
722_WM_NB_OFF	Wigglematch	1631 – 1518	
881_OS_NB	Ordered Sequence	1627 – 1520	
881_OS_NB_OFF	Ordered Sequence	1620 – 1516	
882_OS_NB	Ordered Sequence	1672 – 1533	(1672 – 1652, 1645 – 1533)
882_OS_NB_OFF	Ordered Sequence	1627 – 1519	
882_OS_WB	Ordered Sequence	1610 – 1510	
882_OS_WB_OFF	Ordered Sequence	1602 – 1502	
882_UN_NB	25% Uncertainty	1666 – 1534	(1666 – 1657, 1640 – 1534)
882_UN_NB_OFF	25% Uncertainty	1624 – 1529	
882_UN_WB	25% Uncertainty	1616 – 1534	
882_UN_WB_OFF	25% Uncertainty	1729 – 1522	(1729 – 1714, 1612 – 1522)
883_OS_NB	Ordered Sequence	1606 – 1506	
883_OS_NB_OFF	Ordered Sequence	1601 – 1501	
883_UN_NB	25% Uncertainty	1614 – 1520	
883_UN_NB_OFF	25% Uncertainty	1631 – 1519	
883_WM_NB	Wigglematch	1615 – 1527	
883_WM_NB_OFF	Wigglematch	1611 – 1509	

Supplementary Methods

M1: Details of off-set justification: The large juniper data set was run over a c.6 month period in random, non-sequential order (ignoring calendar age) at the University of Arizona radiocarbon laboratory. Samples were run a few to each wheel, interspersed with a variety of other samples not connected to this work. The first laboratory number (AA) assigned to the juniper group was AA108707, the last was AA111568. The Therasia samples were run in two groups within the time period bracketed by these AA numbers, Therasia AA110271-5 towards the start of the analytical period, and AA111456-9 towards the end of the period. The off-set of the juniper samples relative to IntCal20 throughout the calendar time-period shown in Fig. 4 in the main text is consistent and would seem most likely to reflect the difference in a data-set from a single laboratory source during a given time period and the position of the combined IntCal calibration curve (which incorporates data from a number of growth regions and laboratories) for this same period. On this basis, as the olive samples which are from the same latitude and growth region as the juniper trees and were run within this group of samples which show a consistent off-set we can there for fine tune calibration accordingly. We note that we know the growth region specific differences between these data and oak and pine data that are actually included in IntCal. These were previously published in Pearson et al 2020. The junipers, measured at the same laboratory during the same analytical period as the oak and pine trees also were shown to produce radiocarbon measurements +9.0 ± 3.5 ^{14}C years older than the Irish oaks and +3.4 ± 2 ^{14}C years older than contemporaneous North American bristlecone pine. These differences must contribute in part to the off-set now observed between the juniper data and IntCal20.

M2: Details of off-set calculation:

In order to estimate the posterior for DeltaR for the juniper trees, the raw juniper data published in Pearson et. al 2020 were used as follows in R:

```
# Read in the juniper trees

Juniper_Temp <- read.csv("JuniperRawData.csv", header = TRUE)


# Clean up the NAs (which are the outliers)

Juniper_Data <- Juniper_Temp[, c("INTCALBP", "GOR14Cage", "error")]

Juniper_Data <- Juniper_Data[complete.cases(Juniper_Data),]

rm(Juniper_Temp)

IntCal20 <- read.table("intcal20.14c", sep = ",",

                    header=FALSE, skip=11)

names(IntCal20) <- c("calage", "c14age", "c14sig", "D14C", "D14Csig")
```

M3: Details of modeling choice:

There are a large number of variations to use for modeling these data based on previous published works. In this study we favor a 'simple ordered sequence' as used in Pearson et al. 2018 because this begins with a premise that is routed in fact and does not make any assumptions (we can be certain the pith sample is unquestionably older than the outermost edge sample). The simple ordered sequence model does not include boundaries (either growth counts or within the OxCal code), it simply takes the oldest part of the shrub and the youngest part of the shrub and matches these to the calibration curve. Table S1, figures S1,2,3 and 4 show a range of modeled possibilities for the Therasia olive ranging from most conservative to more speculative / subjective. Specific notes for each sample are included with Figures S1-4. Overall the data demonstrate a range of possible age estimates for the shrub as a whole with no single, certain 'correct answer', however probability distributions overall appear to more strongly align with known dates for volcanic eruptions revealed in the ice cores in the mid-later part of the 16th century BCE than with those currently known around 1600 BCE.

Code example:

Simple sequence with Delta_R

```
Plot()
{
  Delta_R("Offset",13.7, 2);
  Sequence()
  {
    R_Date("88-1 I",3398,21);
    R_Date("88-1 O",3320,22);
  }
};
```

Figure S1: Extended modeling possibilities for Therasia Olive sub-sample 77-2. This sample had an intact outer edge which may or may not represent time of death according to what is known about olive growth. It may, but given the younger radiocarbon dates derived from other parts of the shrub, it also may not. There is uncertainty. This sub-sample had the best preserved growth structures however. In several places the bands were clearly defined and may represent annual growth, in others they were less distinguishable. Uncorrected, each of the modeling attempts produce similar probability density distributions for this sample which could support a death date for the shrub in either 1611 BCE or 1562-1555 BC (evident in the ice core record). Fine-tuning the result to intCal using the offset correction changes the end date to support an association with volcanic events at 1562-1555 BCE or after depending on whether the outer-edge measurement represents time of death.

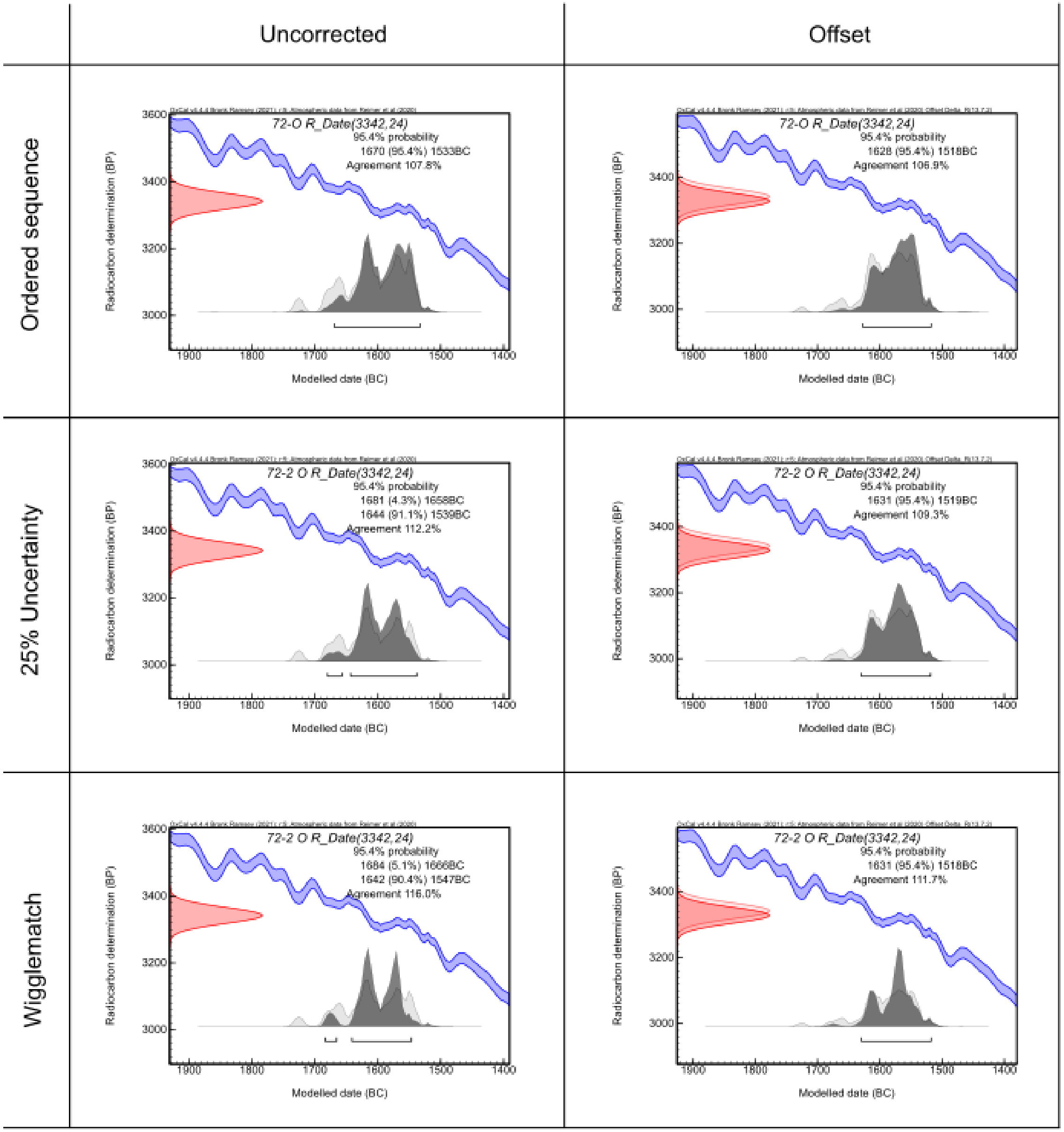

Figure S2: Extended modeling possibilities for Therasia Olive sub-sample 88-1. This sample had a possible growth hiatus identified in the anatomical structure and then supported by the radiocarbon results. Growth boundaries were unclear and impossible to count in several areas. We therefore did not attempt to use growth band counts in this case even for experimental modeling. As such we regard this sample as less reliable in terms of an end date for the shrub, however the innermost portion of the sample represents the earliest terminus post quem (same uncertainties applying) provided by the shrub as a whole for the first earthquake destruction evident on Therasia and Thera in the years leading up to the eruption. It was raised in review that this date may also be a slight analytical outlier given its difference from the rest of the shrub pith dates. While we cannot wholly rule this out without re-running the analysis, we note that the calibrated result is consistent with others from similar contexts.

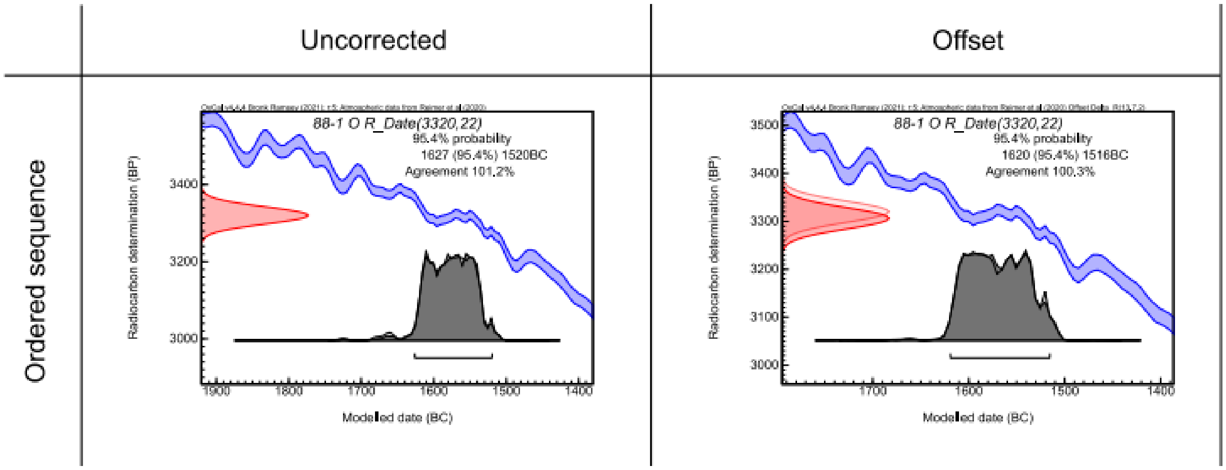

Figure S3: Extended modeling possibilities for Therasia Olive sub-sample 88-2. Sample 88-2 had intact, carbonized bark which was also prized off the main stem for separate radiocarbon analysis, we opted to present the simple ordered sequence for the sample as a whole with and without bark in the main text. 'Without bark' recognizes the fact that we cannot fully assume that the bark sample is all younger than the outer stem, as the bark may store ^{14}C over a number of years in lower layers while still photosynthesizing in the external layers at time of death. In view of the fact that the radiocarbon determination for the bark is a measurement which falls clearly on the radiocarbon plateau, if a number of years were included you would expect them all to have a similar radiocarbon measurement and not impact the end result. More importantly if the bark included radiocarbon from years that were older than the outer edge you would expect it to have the same radiocarbon determination or older than the outer edge. As it does not and the calibrated result is almost identical to that of the small twig with intact juvenile bark at the time of carbonation (88-3), this suggests the bark is in fact truly younger than the outermost edge of sample 88-2 and we feel justified in highlighting the ordered sequence including the bark. The 25% uncertainty model using approximate growth band counts with and without bark is also shown corrected and uncorrected. Counts in this sample were possible in most places, but bands were ultra fine and counts on different radii differed. All scenarios present heightened probability density distributions in the 16th century, marginalizing the possibility of an association with the ice core acidity at 1611 BCE.

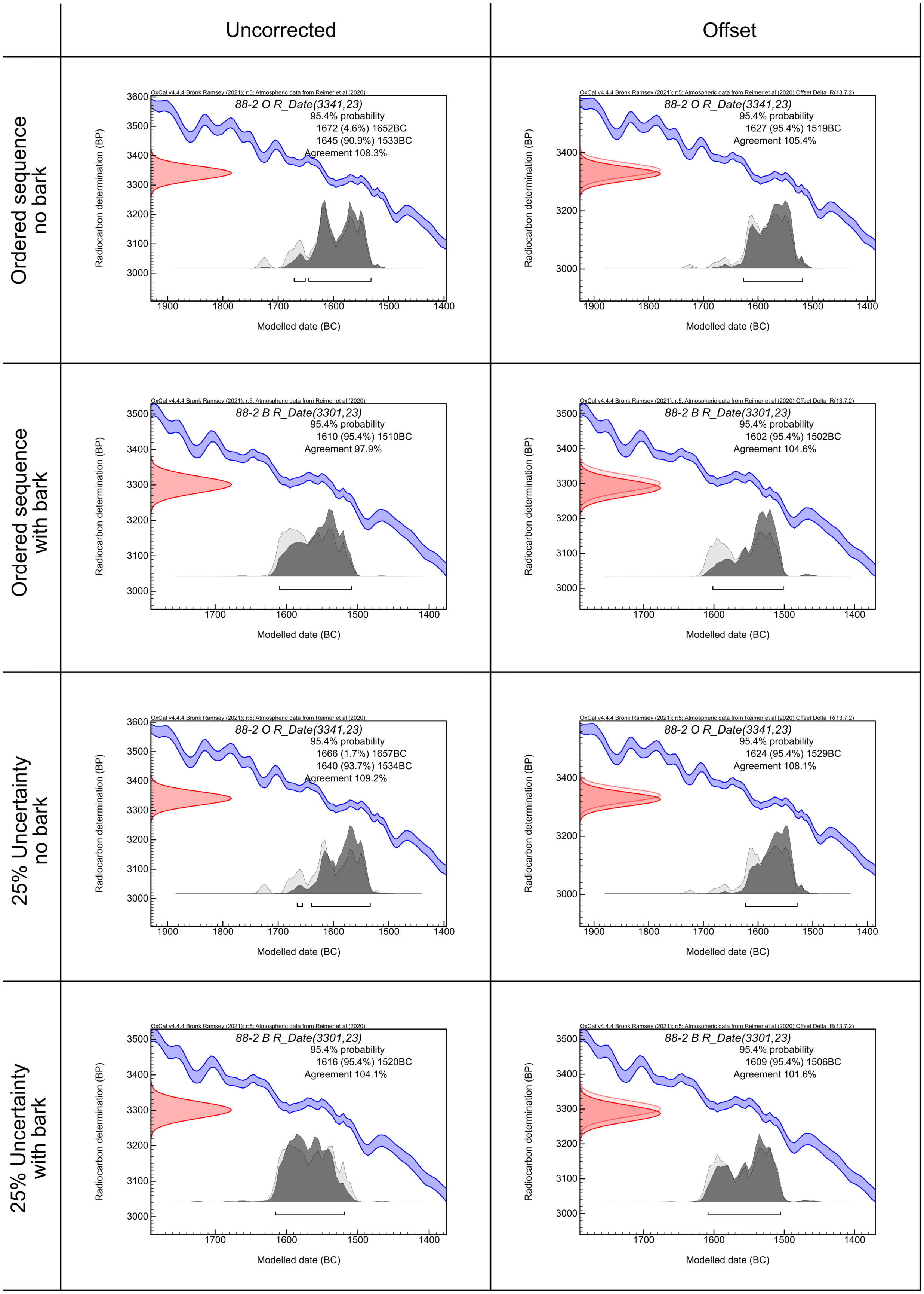
Uncorrected
Offset
Ordered sequence no bark
Ordered sequence with bark
25% Uncertainty no bark
25% Uncertainty with bark
OxCal v4.4.4 Bronk Ramsey (2021); r:5; Atmospheric data from Reimer et al (2020)
88-2 O R_Date(3341,23)
95.4% probability
1672 (4.6%) 1652BC
1645 (90.9%) 1533BC
Agreement 108.3%
OxCal v4.4.4 Bronk Ramsey (2021); r:5; Atmospheric data from Reimer et al (2020) Offset Delta_R(13,7.2)
88-2 O R_Date(3341,23)
95.4% probability
1627 (95.4%) 1519BC
Agreement 105.4%
88-2 B R_Date(3301,23)
95.4% probability
1610 (95.4%) 1510BC
Agreement 97.9%
88-2 B R_Date(3301,23)
95.4% probability
1602 (95.4%) 1502BC
Agreement 104.6%
88-2 O R_Date(3341,23)
95.4% probability
1666 (1.7%) 1657BC
1640 (93.7%) 1534BC
Agreement 109.2%
88-2 O R_Date(3341,23)
95.4% probability
1624 (95.4%) 1529BC
Agreement 108.1%
88-2 B R_Date(3301,23)
95.4% probability
1616 (95.4%) 1520BC
Agreement 104.1%
88-2 B R_Date(3301,23)
95.4% probability
1609 (95.4%) 1506BC
Agreement 101.6%
Radiocarbon determination (BP)
Modelled date (BC)

Figure S4: Modeling possibilities for the Therasia Olive sub-sample 88-3. 88-3 is very short lived (either 3 wide bands or c.10 minute bands – further work may help clarify this), therefore results are heavily impacted by the plateau effect. This small shoot with paper thin bark is considered highly likely to have been growing at the time of death and the results are identical within error of the bark date from 88-2. The difference for 88-2 is that some degree of extension across the plateau can be suggested by the growth band count and the anchoring of the innermost date for the sample prior to 1620 or so BCE.

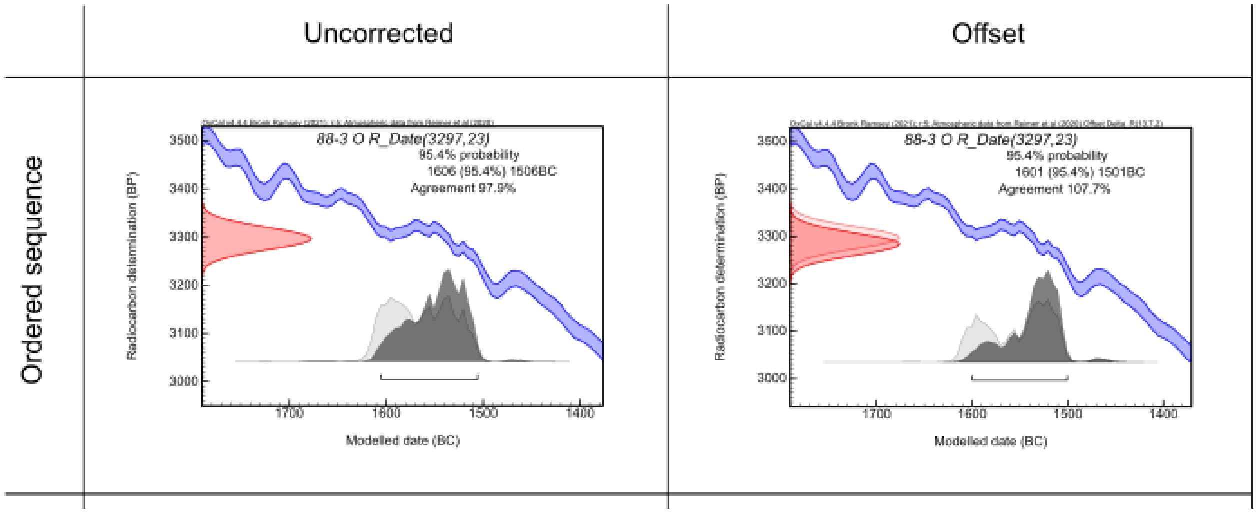

Further work will concentrate on improving the accuracy of the growth band counts via micro CT-Scanning.
